# For patients with non-obstructive azoospermia, the outcome of testicular sperm extraction correlates with self-esteem, sexual health and the quality of the couple’s relationship

**DOI:** 10.1186/s12610-022-00153-z

**Published:** 2022-02-16

**Authors:** Marion BENDAYAN, Emine SAIS, Laura ALTER, Khadija FATHALLAH, Monique JAOUL, Pierre Olivier BOSSET, Geoffroy ROBIN, Florence BOITRELLE

**Affiliations:** 1 Department of Reproductive Biology, Fertility Preservation, Andrology, CECOS, Poissy Hospital, Poissy, France; 2 Paris Saclay University, UVSQ, INRAE, BREED, Jouy-en-Josas, France; 3 Department of Reproductive Medicine, Poissy Hospital, Poissy, France; 4 Department of Urology, Poissy Hospital, Poissy, France; 5 Department of Reproductive Medicine, CHRU de Lille, Lille, France

**Keywords:** Non-obstructive azoospermia, Self-esteem, Sexual disorder, Couple, Relationship, Testicular sperm extraction, Azoospermie non-obstructive, estime de soi, dysfonction sexuelle, relation de couple, biopsie testiculaire

## Abstract

**Background:**

A very small number of studies have indicated that azoospermia or negative testicular sperm extraction (TESE) outcomes are linked to depression or erectile dysfunction. However, the data are often weak, conflicting and gathered with non-validated questionnaires. Hence, we performed a cross-sectional study of 44 men with non-obstructive azoospermia. Levels of self-esteem and the quality of the couple’s sex life and overall relationship were assessed with validated questionnaires before and after the TESE procedure as a function of the TESE outcome.

**Results:**

A positive TESE outcome (*n* = 24) was associated with a statistically significant increase in self-esteem (particularly with regard to family aspects), sexual health and couples’ adjustment quality. In contrast, a negative TESE outcome (*n* = 20) was associated with statistically significant decreases in self-esteem, erectile function, intercourse satisfaction, orgasmic function, couples’ adjustment quality and all aspects of the couple’s relationship (consensus, cohesion, satisfaction and affection).

**Conclusion:**

For men with non-obstructive azoospermia (NOA), negative TESE outcomes may have a negative impact on self-esteem and the quality of the couple’s sex life and overall relationship. This should be borne in mind when counselling men with NOA and their partners to (ideally) help them to cope with and decrease the harmful impacts of azoospermia and negative TESE.

**Supplementary Information:**

The online version contains supplementary material available at 10.1186/s12610-022-00153-z.

## Resume

**Introduction:**

Quelques études concernant l’azoospermie ou les résultats négatifs de l’extraction de sperme testiculaire (TESE) montrent que ces résultats peuvent être liés à la dépression ou aux dysfonctions érectiles. Cependant, les données sont souvent faibles, contradictoires et recueillies à l’aide de questionnaires non validés. Nous avons donc réalisé une étude transversale auprès de 44 hommes atteints d’azoospermie non obstructive (ANO). Les niveaux d’estime de soi et la qualité de la vie sexuelle du couple et de la relation globale ont été évalués à l’aide de questionnaires validés avant et après la procédure TESE en fonction du résultat de la TESE.

**Résultats:**

Un résultat positif de TESE (*n* = 24) a été associé à une augmentation statistiquement significative de l’estime de soi (en particulier en ce qui concerne les aspects familiaux), de la santé sexuelle et de la qualité de l’adaptation du couple. En revanche, un résultat négatif de TESE (*n* = 20) a été associé à une diminution statistiquement significative de l’estime de soi, de la fonction érectile, de la satisfaction des rapports sexuels, de la fonction orgasmique, de la qualité de l’adaptation du couple et de tous les aspects de la relation de couple (consensus, cohésion, satisfaction et affection).

**Conclusion:**

Chez les hommes atteints d’ANO, une TESE négative peut avoir un impact négatif sur l’estime de soi et la qualité de la vie sexuelle et de la relation globale du couple. Il convient de garder cela à l’esprit lors du conseil aux hommes atteints d’ANO et à leurs partenaires afin de les aider (idéalement) à faire face aux impacts néfastes de l’azoospermie et de la TESE négative et à les réduire.

## Introduction

Azoospermia is defined as the absence of spermatozoa in fresh semen after centrifugation of the entire ejaculate. It must be confirmed on at least 2 semen samples 3 months apart [1]. It affects about 1% of men in the general population and 5–15% of infertile men [2, 3]. The diagnosis of azoospermia is no longer always synonymous with sterility. Indeed, ICSI (Intra-Cytoplasmic Sperm Injection) has revolutionized the management of these patients by allowing them to consider biological paternity [4]. Testicular sperm extraction (TESE), coupled with ICSI, has made it possible since 1993 to obtain pregnancies and births in couples in which the man has azoospermia [5].

Azoospermia can be caused by two mechanisms that may be associated. It may be an obstructive mechanism with the presence of an obstacle in the male genital tract; this is called obstructive azoospermia (OA). Azoospermia is called non-obstructive azoospermia (NOA) if the mechanism is not obstructive [6].

The distinction between the two types of azoospermia is now based on a combination of clinical (questioning and physical ex-amination), hormonal, ultrasound, genetic and histological diagnosis [7, 8]. While the chances of surgical sperm extraction are over 90% in OA, they are only about 50% in NOA [9]. Hence, in order to better advise the patient before surgery, it would be ideal to predict the presence or absence of testicular spermatozoa and to predict the sperm retrieval rates (SRR). Unfortunately, no single parameter appears to be sufficiently predictive of the outcome of testicular sperm retrieval (for review see [5, 10–12]), although some scoring criteria have been suggested [13, 14]. Hence, it is difficult to reliably counsel men with NOA on the likelihood of a positive TESE outcome.

Infertility in general (and azoospermia in particular) may impact a couple’s quality of life and relationship quality. Accordingly, the European Society of Human Reproduction and Embryology recently published guidelines on psychosocial care in infertility and medically assisted reproduction [15]. Furthermore, the diagnosis of azoospermia seems to be associated with feelings of shame and guilt towards the partner. Indeed, these are the feelings expressed by a majority of the 441 childless men surveyed in a study about how they would feel if they knew they were azoospermic [16]. There are very few studies regarding the assessment of quality of life and psychological impact after a TESE procedure.

Hence, we hypothesized that a diagnosis of azoospermia could impact a man’s psychological and sexual health and the quality of the relationship with his partner. Furthermore, we hypothesized that the TESE outcome (positive or negative) could also impact these parameters. Given that (i) the outcome of the TESE procedure in NOA cannot be reliably predicted, (ii) azoospermia may have an impact on male self-esteem, sexual health and relationship quality, and (iii) the presence or absence of testicular sperm might also have an impact on these latter parameters, NOA patients’ self-esteem, sex life quality and relationship quality before and after the TESE procedure, as a function of subsequent sperm retrieval (positive vs. negative) were studied.

## Materials and methods

### Patients

Between 2016 and 2020, 124 patients diagnosed with azoospermia were monitored in our centre. Before the TESE procedure, each patient underwent a comprehensive andrology workup to determine the aetiology of azoospermia (medical history, physical examination, seminal markers, ultrasound imaging of the urogenital tract and testes, serum FSH, inhibin B, total testosterone and LH levels, karyotyping and Yq microdeletion analysis), as previously recommended (for a review, see [8, 17]).

**Fig. 1:**
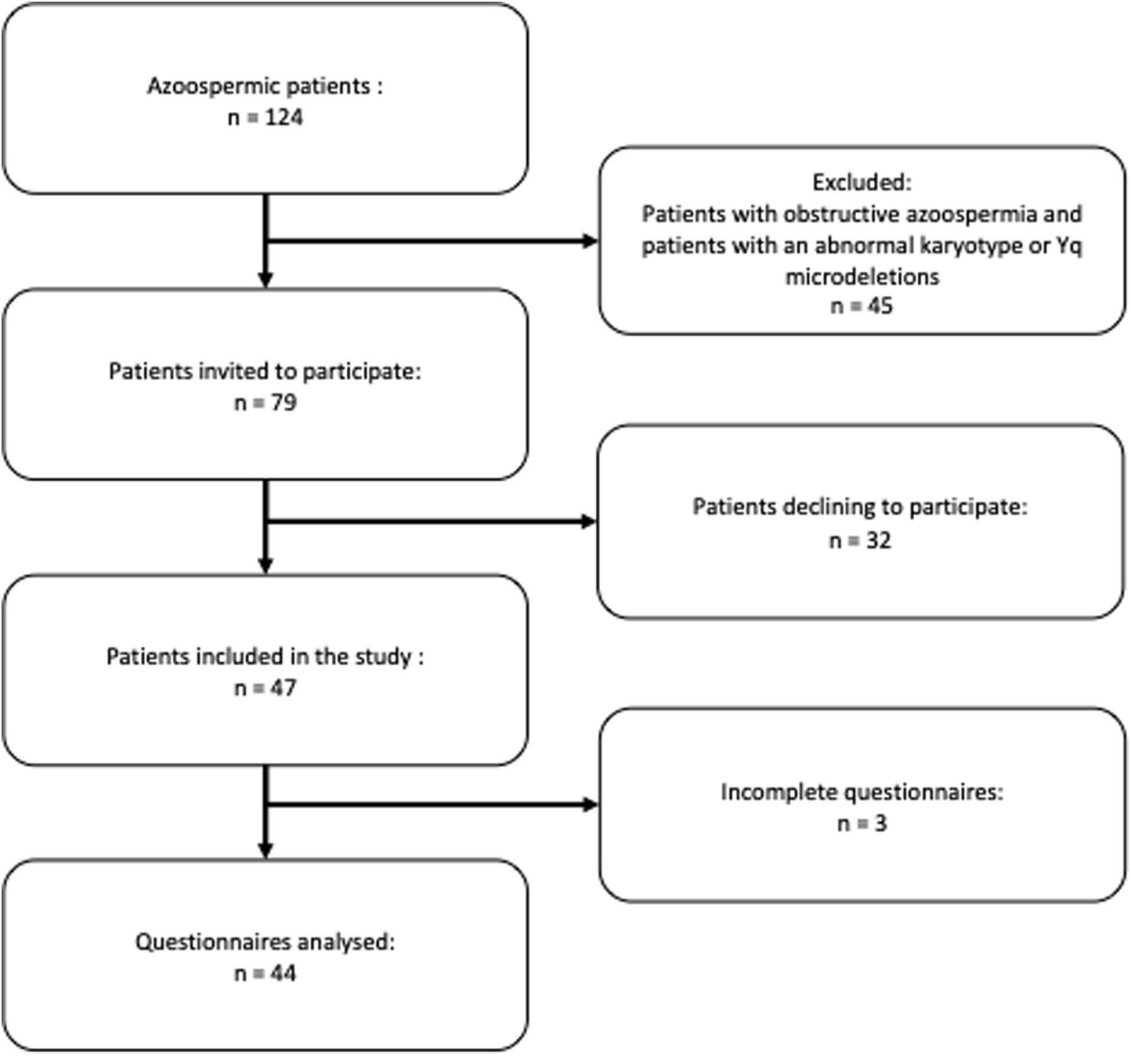
Flow-chart

After the exclusion of patients with OA and patients with an abnormal karyotype or Yq microdeletions (*n* = 45), 79 patients were invited to participate in the study. Thirty-two declined, and therefore 47 men were included in the study. Among the patients who refused to participate, multiple reasons were given. Some patients had planned to perform the TESE procedure at another hospital. Some did not wish to complete any of the questionnaires. Finally, some patients had just been diagnosed with azoospermia, were unsure whether they wanted a TESE procedure and did not wish to participate in our study. Three men did not complete the post-TESE evaluation. Hence, pre- and post-TESE data were analysed for a total of 44 patients with NOA (Fig. 1).

### TESE

For patients who had undergone TESE, a TESE was considered positive if sperm were found and considered suitable for ICSI. The patient was then informed that the TESE was positive and that intra-conjugal ART could be performed. A TESE was considered negative in the opposite case.

### Questionnaires

All patients were interviewed in our fertility centre by the same trained physician three months before and three months after the TESE procedure.

During the interview, the three validated study questionnaires were presented to the patients, and a total testosterone assay was performed (correcting for bias due to hypogonadism). At the end of the interview, the patient was invited to participate in the study. Then, after reading the study information sheet and signing the consent form, the participant completed the three study questionnaires in a separate room, in the presence of an investigator. The study questionnaires were the Coopersmith Self-Esteem Inventory (SEI) [18], the 15-item International Index of Erectile Function (IIEF-15) [19, 20], and the Dyadic Adjustment Scale (DAS) [21]. All three questionnaires have been extensively validated in the literature.

#### The Coopersmith self esteem inventory

The 58-item SEI is one of the most frequently applied measures of self-esteem (positive and negative attitudes toward oneself). It was originally designed for children but was subsequently modified to be compatible for adults. Coopersmith considered that self-esteem expresses (i) approval or disapproval of oneself, (ii) the extent to which one believes that one is talented or successful, and (iii) the meaning and value of one’s life. For each of the 58 items, the participant must indicate whether the corresponding statement is “like me” or “unlike me”. The SEI assesses personal self-esteem (26 items), social self-esteem (8 items), professional self-esteem (8 items) and family-related self-esteem (8 items). The test has a built-in 8-item “lie scale” to help to determine whether the responder is trying too hard to appear to have high self-esteem. If the participant answered “like me” to three or more of these eight items, he had to retake the test with an eye toward being more realistic in his responses. The results were assessed using the following validated criteria; 47 < SEI < 50: significantly above-average self-esteem; 40 < SEI ≤ 47: somewhat above-average self-esteem; 40: average self-esteem; 36 < SEI < 40: somewhat below-average self-esteem; 33 < SEI ≤ 36: significantly below-average self-esteem; 18 < SEI ≤ 33: low self-esteem; SEI ≤ 18: very low self-esteem. The maximum possible score for personal subscore is 26. The maximum possible score for each other subscore (social, professional and family-related self-esteem) is 8; the higher the score, the higher the self-esteem.

#### The IIEF-15

The IIEF-15 is often applied in the field of urology and andrology (Supplemental Table 1). We decided to administer Version 1 of the IIEF-15, which is more applicable to heterosexual men. The IIEF-15 assess erectile function (6 items), orgasmic function (2 items), sexual desire (2 items), intercourse satisfaction (3 items) and overall satisfaction (2 items). The maximum possible total score is 75, with a maximum score of 30 for erectile function, 15 for sexual intercourse satisfaction, and 10 for each of the orgasmic function, sexual desire and overall satisfaction domains. For erectile function, the score is interpreted as follows; 1–10: severe erectile dysfunction; 11–16: moderate dysfunction; 17–21: mild-to-moderate dysfunction; 22–25: mild dysfunction; 26–30: no dysfunction. In the other domains, higher scores also correspond to less dysfunction.

#### The DAS

The 32-item DAS self-questionnaire is one of the best instruments for measuring a couple’s dyadic relationship. It displays concurrent and predictive validity and has been cited more than 6000 times in PubMed. The DAS score varies as a function of personal circumstances and life events; hence, this scale enabled us to assess and compare the levels of dyadic adjustment before and after TESE in azoospermic men. The DAS produces an overall score (maximum: 151) and a score for each of the four following subscales: dyadic consensus (12 items and a maximum score of 60), dyadic satisfaction (6 items and a maximum score of 31), dyadic cohesion (10 items and a maximum score of 48), and affection expression (4 items and a maximum score of 12 points) [21]. Lower scores are known to be associated with an increased likelihood of domestic violence, depression, misunderstanding, and poor communication. In general, a DAS total score above 100 testifies to good levels of dyadic adjustment, understanding and communication.

The DAS is a questionnaire used to assess couple relationships. It has been used in the context of infertility in several publications to assess couples on ART or infertile women [22–26].

### Total testosterone assay

Total testosterone was evaluated by an electrochemiluminescence technique. Total testosterone was assayed before 10 am on the day when the patient filled out the questionnaires. Total testosterone values were obtained three months before and three months after TESE. Reference values of the assay were 2.4–8.7 ng/mL.

### Statistical analysis

Data are quoted as the mean ± standard error of the mean (SEM). All statistical analyses were performed with SAS software (version 9.1, SAS Institute Inc., NC, USA). A Wilcoxon rank sum test was used to compare the patients’ data before and after the TESE procedure. In all tests, the threshold for statistical significance was set to *p* ≤ 0.05.

## Results

### Self-esteem

**Fig. 2:**
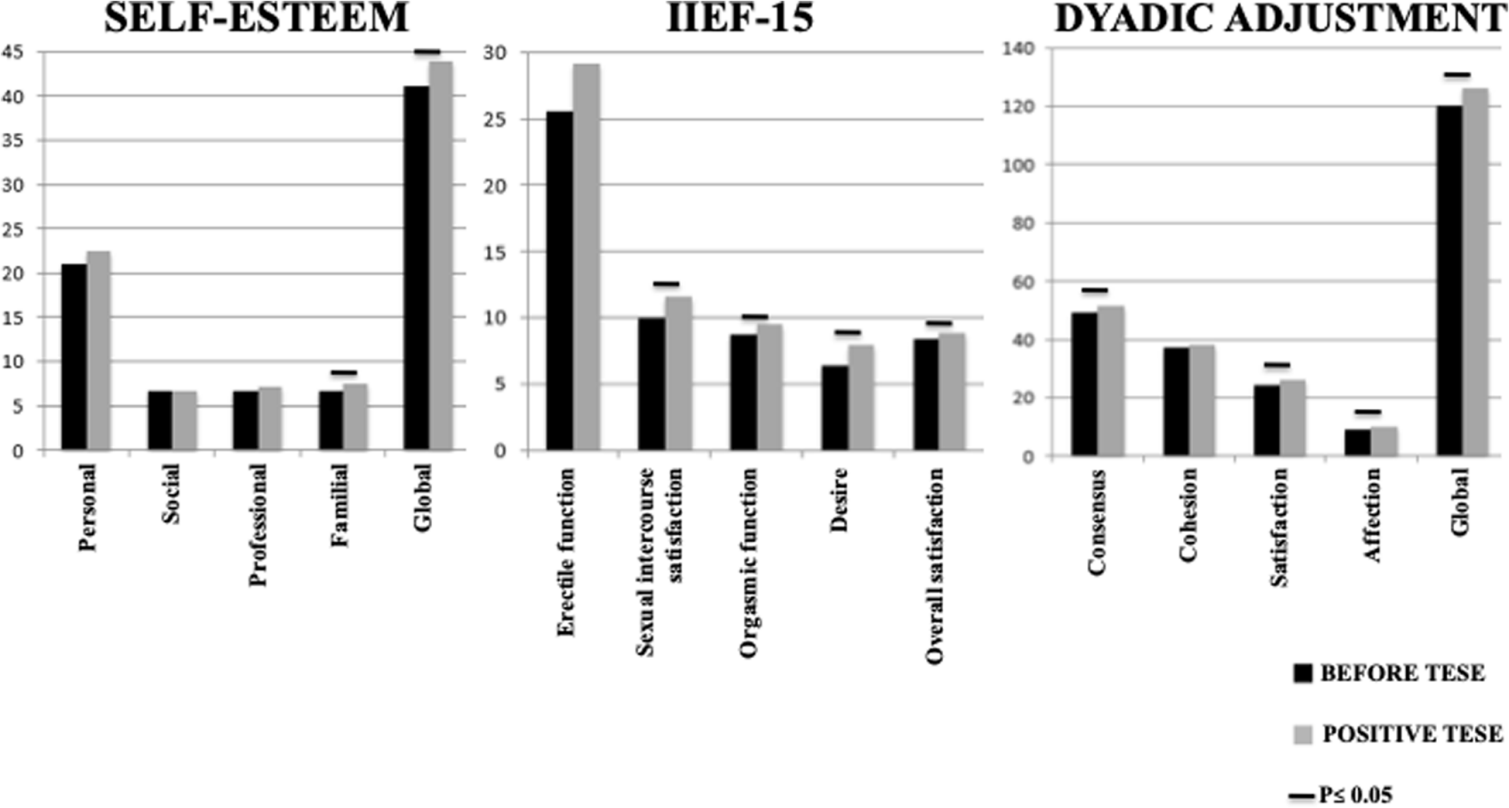
Self-Esteem, Sexual Life Quality and Dyadic Adjustment before TESE and after positive TESE (*n* = 24). *TESE: Testicular sperm extraction. IIEF-15: 15-item International Index of Erectile Function. P value was calculated using a Wilcoxon test. P < 0.05 was considered as significant*

**Fig. 3:**
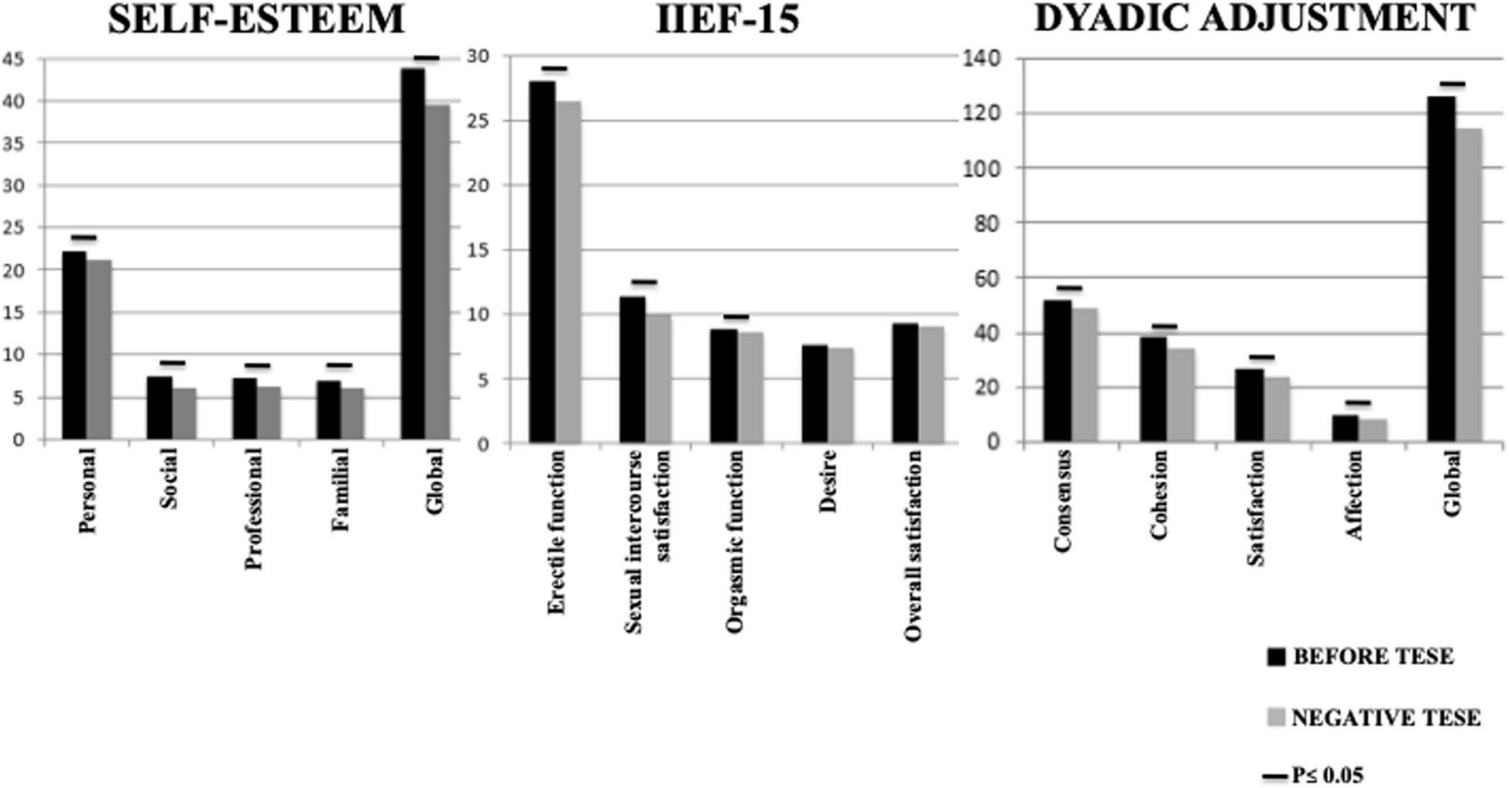
Self-Esteem, Sexual Life Quality and Dyadic Adjustment before TESE and after negative TESE (*n* = 20). *TESE: Testicular sperm extraction. IIEF-15: 15-item International Index of Erectile Function. P value was calculated using a Wilcoxon test. P < 0.05 was considered as significant*

Overall, the 44 patients had slightly higher than average levels of self-esteem before and after the TESE procedure, when looking at the score as a whole and taking all patients into account. Self-esteem levels differed significantly in function of TESE outcome. Patients with a positive TESE outcome (*n* = 24) had a significantly higher mean level of overall self-esteem after the TESE procedure (44.0 ± 1.2) than before the procedure (41.1 ± 2.0; *p* = 0.01). This was essentially due to a significant increase in family-related self-esteem (7.5 ± 0.2 after TESE vs. 6.7 ± 0.3 before TESE; *p* = 0.002). This was the only significant pre−/post- difference in self-esteem for participants with a positive TESE outcome (Fig. 2). In contrast, patients with a negative TESE outcome (*n* = 20) had a significantly lower level of overall self-esteem after TESE than before (39.5 ± 0.9 vs. 43.8 ± 0.7, respectively; *p* < 0.001). This significant decrease in self-esteem after a negative TESE outcome was observed in all SEI domains: personal self-esteem (21.1 ± 0.5 after TESE vs. 22.2 ± 0.3 before; *p* < 0.01), social self-esteem (6.1 ± 0.3 vs. 7.4 ± 0.2, respectively; p < 0.01), professional self-esteem (6.2 ± 0.4 vs. 7.3 ± 0.2, respectively; p < 0.01) and family-related self-esteem (6.1 ± 0.4 vs. 6.9 ± 0.4, respectively; p < 0.01) (Fig. 3).

### Sexual health

#### Erectile function

Overall, the 44 patients obtained a good score of erectile function before and after the TESE procedure. Patients with a positive TESE outcome had a higher level of erectile function after the TESE procedure than before, although the difference was not quite statistically significant (*p* = 0.051; Fig. 2). Patients with a negative TESE outcome had a significantly worse erectile function after the TESE procedure than before (26.5 ± 0.9 vs. 28.0 ± 0.7; *p* = 0.04; Fig. 3).

#### Other sexual functions

In general, the participants obtained a good score of intercourse satisfaction, orgasmic function, desire and overall satisfaction both before and after the TESE procedure. However, several sexual function scores differed significantly as a function of the TESE outcome. Patients with a positive TESE outcome had significantly higher levels of intercourse satisfaction, orgasmic function, sexual desire and overall satisfaction after the TESE procedure than before (Fig. 2). In contrast, patients with a negative TESE outcome had significantly lower levels of sexual intercourse satisfaction and orgasmic function after the TESE procedure than before (Fig. 3).

#### Relationship quality, as assessed by the DAS

In general, the 44 participants obtained a good score of dyadic adjustment before and after the TESE procedure. However, the DAS scores differed as a function of TESE outcomes. Indeed, patients with a positive TESE outcome had a significantly higher overall score after the TESE procedure than before (126.3 ± 2.5 vs. 120.3 ± 3.2, respectively; *p* < 0.001) (Fig. 2). Their consensus, satisfaction and affection subscores were also significantly higher after the procedure (Fig. 2). In contrast, patients with a negative TESE outcome had significantly lower scores after the TESE procedure than before (114.7 ± 3.0 vs. 126.8 ± 1.6, respectively; *p* < 0.0001). Likewise, all the DAS subscores were significantly lower after the procedure (Fig. 3).

#### Total testosterone levels

For the study population, the mean serum total testosterone levels were significantly lower three months after TESE than before (3.9 ± 1,2 ng/ml vs. 5.2 ± 0.7 ng/ml, respectively p < 0.0001) (Supplemental Table 2). The decrease did not differ as a function of TESE outcomes. More precisely, 28 of the 44 patients had a below-average serum total testosterone level after the TESE procedure. For the 10 patients with lower self-esteem after the TESE procedure, only two had a below-average serum total testosterone level. Similarly, only six of the 18 men presenting with alterations in one or more sexual functions after the TESE procedure had a below-average testosterone level. Lastly, no patients with worse dyadic adjustment after the TESE procedure had a below-average testosterone level.

## Discussion

Searching the PubMed database between February 1965 and November 2021 with the search terms “azoospermia” and (“psychological” or “self-esteem” or “erecti*” or “marital”) produced 73, 5, 85 and 40 records, respectively. None of these studies answered the questions we asked in the present work.

First, we observe a decrease in testosterone levels after the TESE procedure compared to before surgery. This decrease had already been shown in several studies. A study of 435 men diagnosed with azoospermia showed a 20% decrease in testosterone concentration after a TESE procedure compared to the preoperative concentration [27]. Another study of 48 men who had a TESE procedure showed the same results: 31 patients in this study had pre- and postoperative hormone monitoring. Testosterone concentration was 10% lower after the TESE procedure (*p* < 0.05). Five of the 31 patients (16%) developed androgen deficiency postoperatively [28].

In 2020, another study was published concerning 60 patients with NOA, with normal testosterone and LH levels. After the TESE procedure, a significant decrease in testosterone levels and a significant increase in LH levels were shown at 24 h and at one week postoperatively [29]. A review of the literature published in 2021 shows that the TESE procedure can lead to structural disturbances of the testicular tissue leading to an alteration of the vascularization and can result in androgen deficiency [30]. Our results are thus consistent with the data in the literature.

Secondly, in this study of men with NOA, we found that the TESE outcome had a negative correlation with self-esteem, sexual health and couples’ relationship quality.

For the first time, here, self-esteem in men with NOA as a function of the TESE outcome was studied. It has already been shown that the announcement of male infertility could cause the same reactions as that of serious illness: shame, guilt, depression, anxiety and stress [31]. Infertility due to male factors may also decrease the quality of an infertile man’s personal relationships and sexual life (for a review, see [31, 32]). However, very few studies have focused on the feelings of patients diagnosed with azoospermia [33, 34]. Azoospermia reportedly induced anxiety, depression [33] or guilt in men because the treatments they inflicted to their wives [34]. Here, we found that patients with a positive TESE outcome had a higher level of overall self-esteem after TESE than before TESE; this was due solely to a significant increase in family-related self-esteem. Hence, we hypothesize that (i) a diagnosis of azoospermia had a negative impact on the man’s family-related quality of life and (ii) the retrieval of spermatozoa enabled the men to recover some or all of all this family-related self-esteem. In contrast, a negative TESE outcome was accompanied by significant decreases in overall, personal, social, professional and family-related self-esteem. Hence, our present results show that definitive azoospermia had a negative impact on the men’s self-esteem, their belief that they are talented and successful, and their belief that their life has meaning and value (according to Coopersmith). We suggest that psychological follow-up might be of value in assessing and then countering this loss of self-esteem.

Similarly, our review of the literature suggests that the present study is the first to have studied all aspects of sexual health quality in NOA patients as a function of the TESE outcome. We found that men with a positive TESE outcome reported significantly better levels of sexual function and satisfaction after the TESE procedure than before. In contrast, patients with a negative TESE outcome showed lower levels of erectile function, intercourse satisfaction and orgasmic function after TESE than before. Only one previous study has focused on erectile function in men diagnosed with azoospermia as a function of the TESE outcome [35]; the researchers found that men with a negative TESE outcome were more likely to present erectile dysfunction than men with a positive TESE outcome. However, the researchers did not discuss the large decrease in testosterone levels that they observed after TESE; this would have been important because testosterone may impact all these sexual parameters. In the present study, TESE outcomes were found to have an impact on erectile function and all other sexual functions, independently of the serum total testosterone level. In view of the observed relationship between definitive azoospermia and sexuality, our results suggest that the sexual health of men needs to be monitored and managed more efficiently.

Finally, a positive TESE outcome had a positive impact on dyadic adjustment in general and on consensus, satisfaction and affection in particular. A negative TESE outcome was found to negatively impact all aspects of dyadic adjustment and all aspects of the relationship with the partner (consensus, cohesion, satisfaction and affection). Thus, a negative TESE outcome might explain (at least in part) the observed changes in the couples’ relationship, communication and understanding. These results yet again indicate the need for better monitoring and management of intimate relationships in men with definitive azoospermia, in order to help them to express their difficulties and improve levels of communication and understanding within the couple.

In conclusion, definitive azoospermia was found to have a negative impact on many aspects of self-esteem, sexual health and relationship quality. These results suggest the need for better management of men with NOA. These men are frequently lost to follow-up once it has become clear that intra-couple assisted reproductive techniques will not be successful. Some couples give up their efforts to have a child, others turn to adoption procedures, and yet other choose donor sperm programmes. It seems necessary to provide patients with clear information about the expected outcome and the impact it may have on their quality of life prior to the TESE procedure. Psychological and possibly sexological follow-up should be offered to patients after the TESE procedure, even more so when it has failed. Our results indicate that longer follow-up of these patients would be of value in assessing self-esteem, sex life quality and relationship quality and, ideally, in helping them to cope with the negative impact of azoospermia and failure of TESE. This long-term follow-up of azoosperm patients could indeed help improve their self-image, their quality of sexual life (erectile function, orgasm and sexual satisfaction), and the quality of their relationship. To go further, a psychological, sexological and andrological follow-up was set up in our center at the end of last year. This long-term follow-up of all azoosperm patients will take the form of two interviews at 1 month and 6 months post TESE. This study should allow us to evaluate the interest of a multicentric study on this subject, which is too little discussed in infertility consultations.

## Supplementary Information

**Additional file 1 Supplemental Table 1:** International index of Erectile Function (IIEF-15) questionnaire

**Additional file 2 Supplemental Table 2:** Value of testosterone level in 44 patients before and after TESE

## Abbreviations

DAS: Dyadic Adjustment Scale
FSH: Follicle Stimulating Hormone
ICSI: Intra-Cytoplasmic Sperm Injection
IIEF-15: 15-item International Index of Erectile Function
LH: Luteizing hormone
NOA: Non obstructive azoospermia
OA: Obstructive azoospermia
SEI: Self-Esteem Inventory
SEM: standard error of the mean
SRR: Sperm retrieval rate
TESE: Testicular sperm extraction

## Declarations

### Competing of interests

None to report.

## References

[1] World Health Organization (2021). WHO laboratory manual for the examination and processing of human semen.

[2] Cocuzza M, Alvarenga C, Pagani R (2013). The epidemiology and etiology of azoospermia. Clinics (Sao Paulo).

[3] Jarow JP, Espeland MA, Lipshultz LI (1989). Evaluation of the azoospermic patient. J Urol.

[4] Palermo G, Joris H, Devroey P, Van Steirteghem AC (1992). Pregnancies after intracytoplasmic injection of single spermatozoon into an oocyte. Lancet..

[5] Corona G, Minhas S, Giwercman A, Bettocchi C, Dinkelman-Smit M, Dohle G, Fusco F, Kadioglou A, Kliesch S, Kopa Z, Krausz C, Pelliccione F, Pizzocaro A, Rassweiler J, Verze P, Vignozzi L, Weidner W, Maggi M, Sofikitis N (2019). Sperm recovery and ICSI outcomes in men with non-obstructive azoospermia: a systematic review and meta-analysis. Hum Reprod Update.

[6] Practice Committee of the American Society for Reproductive Medicine in collaboration with the Society for Male R, Urology (2018). Evaluation of the azoospermic male: a committee opinion. Fertil Steril.

[7] Huyghe E, Boitrelle F, Methorst C, Mieusset R, Ray PF, Akakpo W, Koscinski I, Chalas C, Rives N, Plotton I, Robin G, el Osta R, Hennebicq S, Eustache F, Marcelli F, Lejeune H (2021). AFU and SALF recommendations for the evaluation of male infertility. Prog Urol.

[8] Robin G, Boitrelle F, Leroy X, Peers MC, Marcelli F, Rigot JM, Mitchell V (2010). Assessment of azoospermia and histological evaluation of spermatogenesis. Ann Pathol.

[9] Agarwal A, Baskaran S, Parekh N, Cho CL, Henkel R, Vij S, Arafa M, Panner Selvam MK, Shah R (2021). Male infertility. Lancet..

[10] Arshad MA, Majzoub A, Esteves SC (2020). Predictors of surgical sperm retrieval in non-obstructive azoospermia: summary of current literature. Int Urol Nephrol.

[11] Verheyen G, Popovic-Todorovic B, Tournaye H (2017). Processing and selection of surgically-retrieved sperm for ICSI: a review. Basic Clin Androl.

[12] Zarezadeh R, Fattahi A, Nikanfar S, Oghbaei H, Ahmadi Y, Rastgar Rezaei Y, Nouri M, Dittrich R (2021). Hormonal markers as noninvasive predictors of sperm retrieval in non-obstructive azoospermia. J Assist Reprod Genet.

[13] Bernie AM, Ramasamy R, Schlegel PN (2013). Predictive factors of successful microdissection testicular sperm extraction. Basic Clin Androl.

[14] Boitrelle F, Robin G, Marcelli F, Albert M, Leroy-Martin B, Dewailly D, Rigot JM, Mitchell V (2011). A predictive score for testicular sperm extraction quality and surgical ICSI outcome in non-obstructive azoospermia: a retrospective study. Hum Reprod.

[15] Gameiro S, Boivin J, Dancet E, de Klerk C, Emery M, Lewis-Jones C, Thorn P, van den Broeck U, Venetis C, Verhaak CM, Wischmann T, Vermeulen N (2015). ESHRE guideline: routine psychosocial care in infertility and medically assisted reproduction-a guide for fertility staff. Hum Reprod.

[16] Swierkowski-Blanchard N, Alter L, Salama S, Muratorio C, Bergere M, Jaoul M, Vialard F, Bailly M, Selva J, Boitrelle F (2016). To be or not to be [fertile], that is the question. Basic Clin Androl.

[17] Practice Committee of American Society for Reproductive Medicine in collaboration with Society for Male R, Urology (2008). Evaluation of the azoospermic male. Fertil Steril.

[18] Coopersmith S (1967). The antecedents of self-esteem.

[19] Cappelleri JC, Siegel RL, Osterloh IH, Rosen RC (2000). Relationship between patient self-assessment of erectile function and the erectile function domain of the international index of erectile function. Urology..

[20] Rosen RC, Cappelleri JC, Gendrano N (2002). The international index of erectile function (IIEF): a state-of-the-science review. Int J Impot Res.

[21] Spanier GB (1976). Measuring dyadic adjustment: new scales for assessing the quality of marriage and similar dyads. J Marriage Fam.

[22] Donarelli Z, Lo Coco G, Gullo S, Salerno L, Marino A, Sammartano F, Allegra A (2016). The fertility quality of life questionnaire (FertiQoL) relational subscale: psychometric properties and discriminant validity across gender. Hum Reprod.

[23] Kim JH, Shin HS, Yun EK (2018). A dyadic approach to infertility stress, marital adjustment, and depression on quality of life in infertile couples. J Holist Nurs.

[24] Martins MV, Peterson BD, Almeida V, Mesquita-Guimaraes J, Costa ME (2014). Dyadic dynamics of perceived social support in couples facing infertility. Hum Reprod.

[25] Zurlo MC, Cattaneo Della Volta MF, Vallone F. The association between stressful life events and perceived quality of life among women attending infertility treatments: the moderating role of coping strategies and perceived couple's dyadic adjustment (2019). BMC Public Health.

[26] Zurlo MC (2020). Cattaneo Della Volta MF, Vallone F. infertility-related stress and psychological health outcomes in infertile couples undergoing medical treatments: testing a multi-dimensional model. J Clin Psychol Med Settings.

[27] Ramasamy R, Yagan N, Schlegel PN (2005). Structural and functional changes to the testis after conventional versus microdissection testicular sperm extraction. Urology..

[28] Everaert K, De Croo I, Kerckhaert W, Dekuyper P, Dhont M, Van der Elst J (2006). Long term effects of micro-surgical testicular sperm extraction on androgen status in patients with non obstructive azoospermia. BMC Urol.

[29] Sertkaya Z, Tokuc E, Ozkaya F, Ertas K, Kutluhan MA, Culha MG (2020). Acute effect of microdissection testicular sperm extraction on blood total testosterone and luteinising hormone levels. Andrologia..

[30] Billa E, Kanakis GA, Goulis DG. Endocrine Follow-Up of Men with Non-Obstructive Azoospermia Following Testicular Sperm Extraction. J Clin Med. 2021;10(15). 10.3390/jcm10153323 PMC8347935 34362107

[31] Chachamovich JR, Chachamovich E, Ezer H, Fleck MP, Knauth D, Passos EP (2010). Investigating quality of life and health-related quality of life in infertility: a systematic review. J Psychosom Obstet Gynaecol.

[32] Wischmann T, Thorn P (2013). (male) infertility: what does it mean to men? New evidence from quantitative and qualitative studies. Reprod Biomed Online.

[33] Bak CW, Seok HH, Song SH, Kim ES, Her YS, Yoon TK (2012). Hormonal imbalances and psychological scars left behind in infertile men. J Androl.

[34] Johansson M, Hellstrom AL, Berg M (2011). Severe male infertility after failed ICSI treatment--a phenomenological study of men's experiences. Reprod Health.

[35] Akbal C, Mangir N, Tavukcu HH, Ozgur O, Simsek F (2010). Effect of testicular sperm extraction outcome on sexual function in patients with male factor infertility. Urology..

