# Supplementary figures and images for: For patients with non-obstructive azoospermia, the outcome of testicular sperm extraction correlates with self-esteem, sexual health and the quality of the couple’s relationship
Source: Basic Clin Androl. 2022 Feb 16;32:3. doi: 10.1186/s12610-022-00153-z (PMC8848963; doi:10.1186/s12610-022-00153-z)

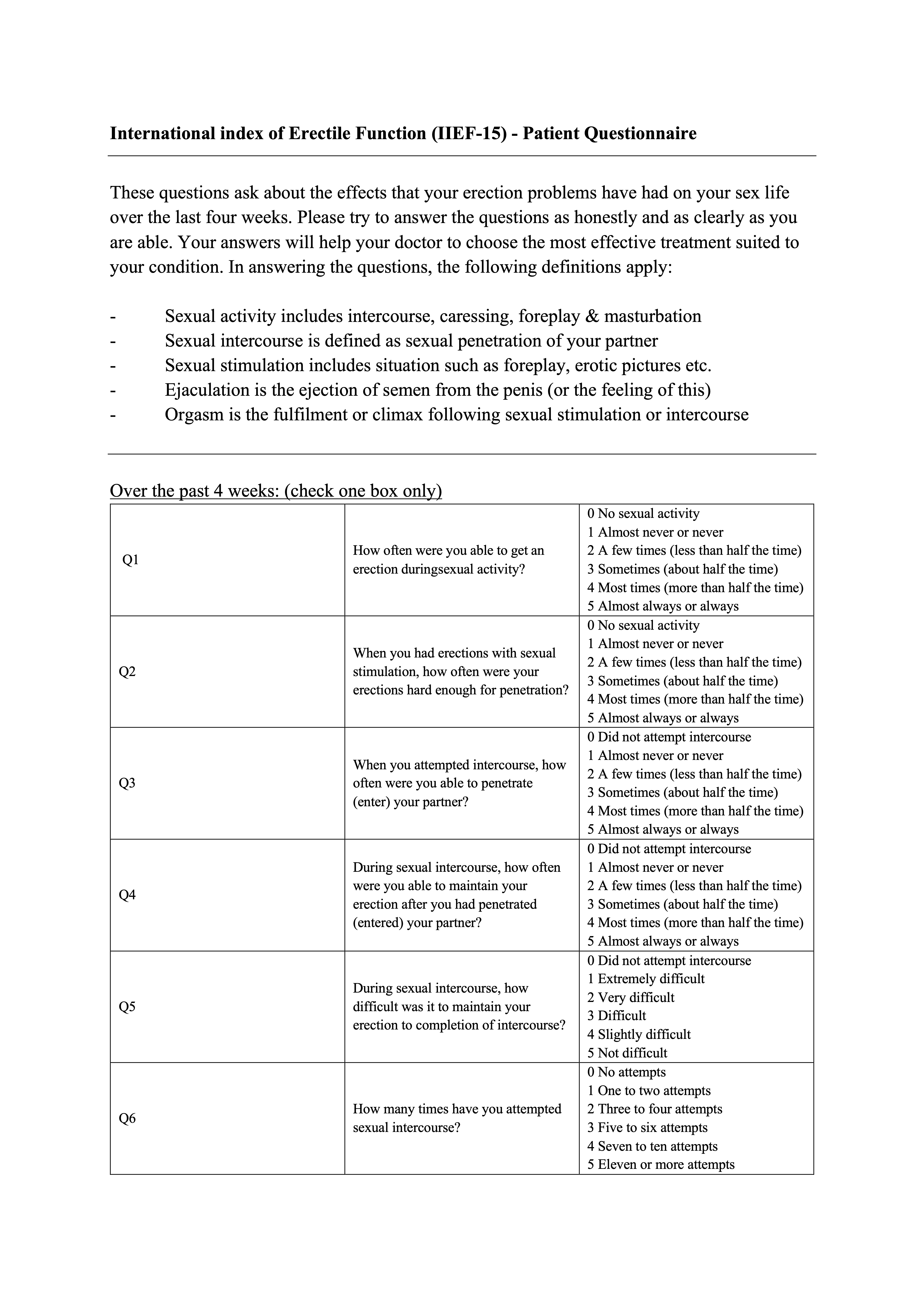

Supplement: Supplementary file 1 — Additional file 1 Supplemental Table 1: International index of Erectile Function (IIEF-15) questionnaire [file 12610_2022_153_MOESM1_ESM.png]

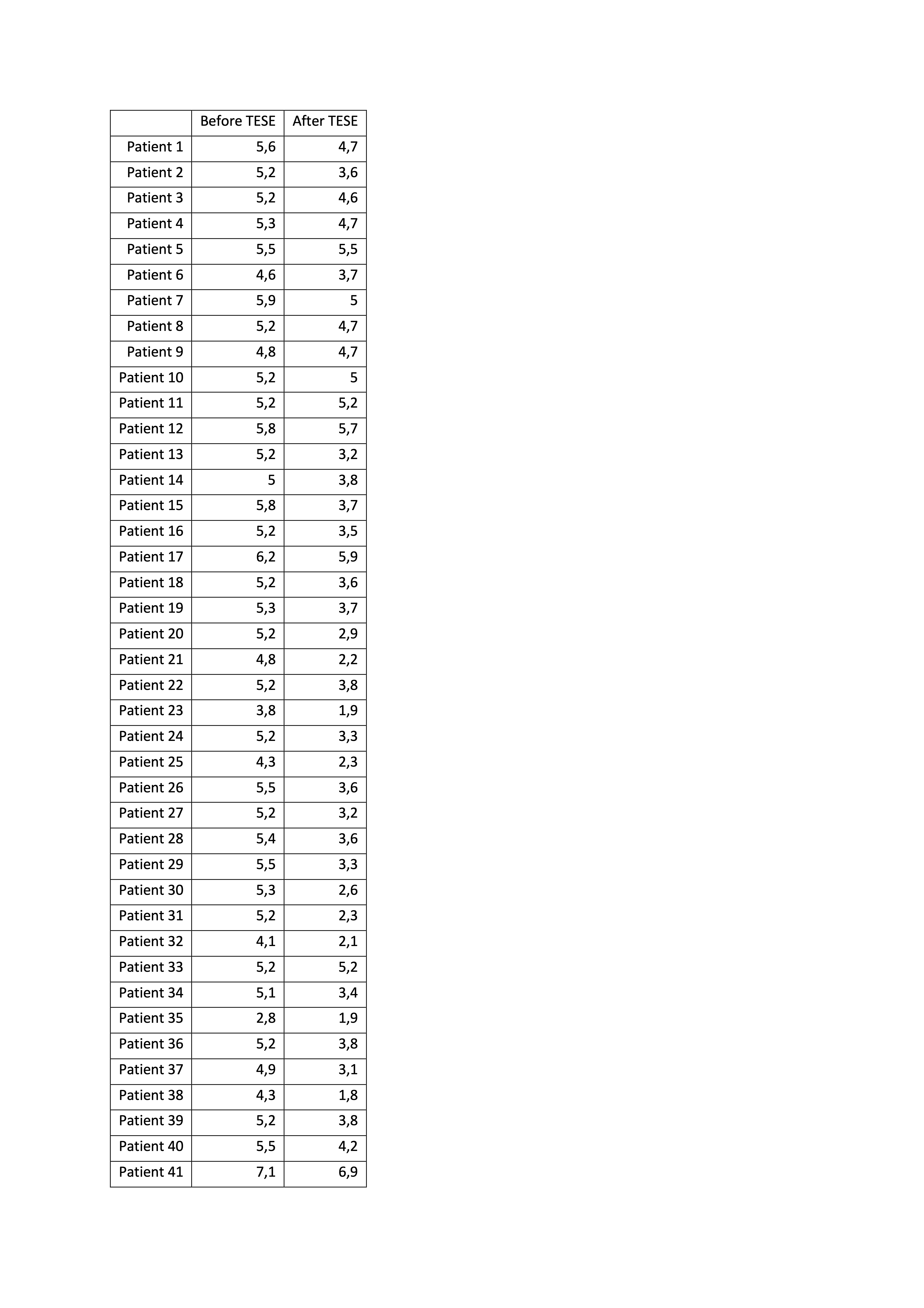

Supplement: Supplementary file 2 — Additional file 2 Supplemental Table 2: Value of testosterone level in 44 patients before and after TESE [file 12610_2022_153_MOESM2_ESM.png]
